# Four-dimensional flow evaluation of left atrial myxoma-related mitral inflow

**DOI:** 10.1093/ehjcr/ytad298

**Published:** 2023-07-06

**Authors:** Hosamadin Assadi, Jordi Broncano, Daniel Carrasco Fernández, Pankaj Garg

**Affiliations:** Norwich Medical School, University of East Anglia, Norwich Research Park, Norwich, Norfolk, NR4 7UQ, UK; Norfolk and Norwich University Hospitals NHS Foundation Trust, Colney Lane, Norwich, Norfolk, NR4 7UY, UK; Cardiothoracic Imaging Unit, Hospital San Juan de Dios, HT Medica, Avenida del Brillante, 106, 14012 Córdoba, Spain; Servicio de Anatomía Patológica, Hospital Universitario Puerta del Mar, Avenida Ana de Viya, 21, 11009 Cadiz, Spain; Norwich Medical School, University of East Anglia, Norwich Research Park, Norwich, Norfolk, NR4 7UQ, UK; Norfolk and Norwich University Hospitals NHS Foundation Trust, Colney Lane, Norwich, Norfolk, NR4 7UY, UK

## Case description

A 71-year-old woman with a history of endometrial cancer was referred for CMR imaging for precise assessment of left ventricular (LV) function after the surgical removal and pharmacological treatment of the tumour. The patient was asymptomatic. The CMR protocol included cines, T1 mapping, T2 mapping, first-pass perfusion, late gadolinium enhancement, and 4D flow imaging. The LV was normal in size (mass, 52 g/m^2^; indexed end-diastolic volume: 66 mL/m^2^) with preserved ejection fraction (60%). The left atrium had a mobile intracavitary mass (32 × 33 × 26 mm; AP × transverse × craniocaudal) with dynamic mitral valve stenosis. The mass was mildly heterogeneous and predominantly hyperintense on T2 short-inversion-time inversion-recovery and T1-weighted images.

**Figure 1:**
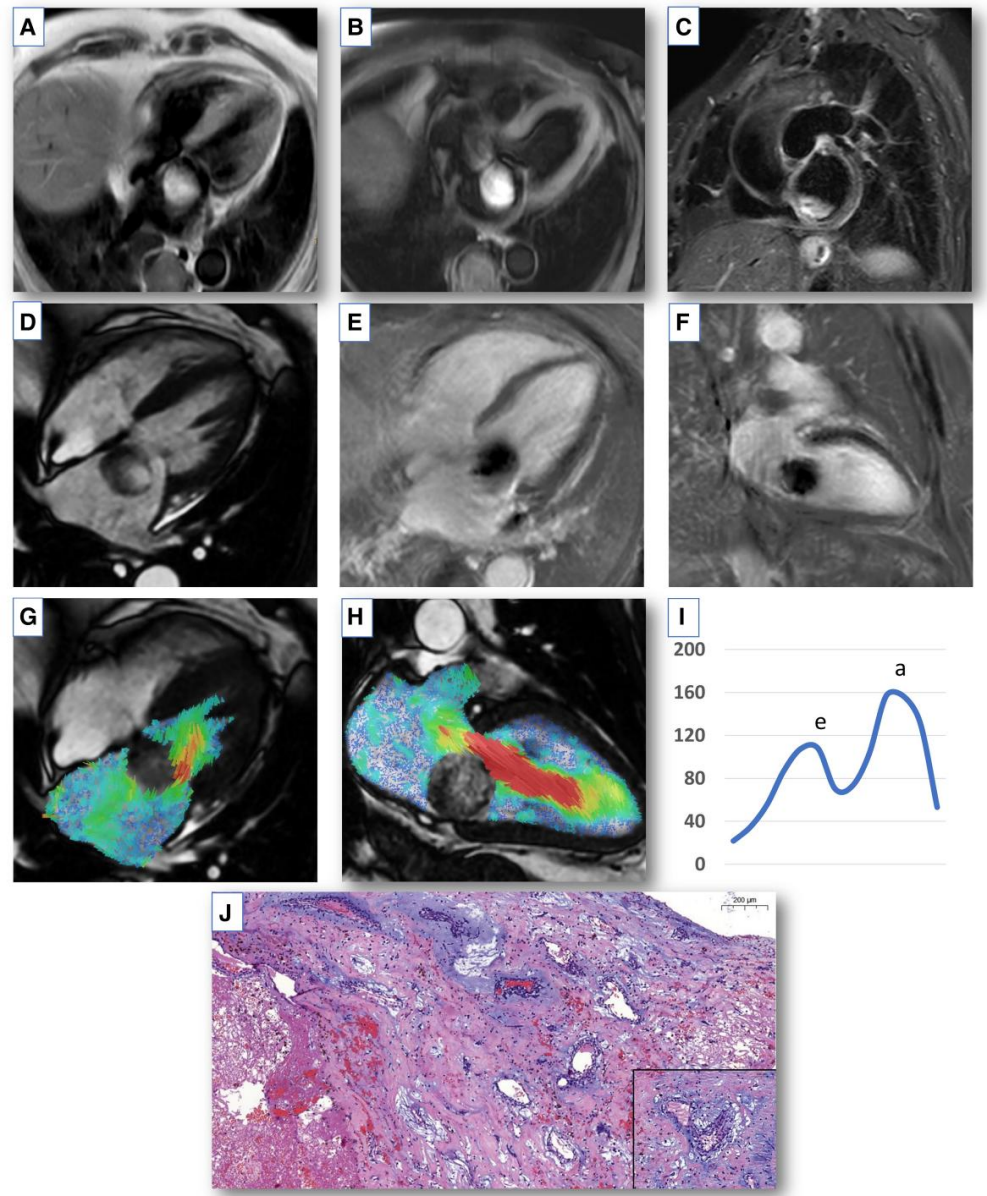
Multi-parametric cardiovascular magnetic resonance imaging of myxoma. (*A*) T1-weighted black pool turbo spin-echo sequences demonstrating a mildly heterogeneous and predominantly hyperintense mass in the left atrium. (*B*) T1-weighted turbo spin-echo with selective fat suppression sequence reveals a hyperintense intracavitary left atrial mass denoting no fat content. (*C*) T2 short-inversion-time inversion-recovery black pool mapping showing a hyperintense and mildly heterogeneous left atrial myxoma. (*D*) Four-chamber cine steady-state free procession cine image showing a mobile intracavitary mass measuring 32 × 33 × 26 mm in the left atrium with heterogenous signal intensity. (*E* and *F*) Inversion recovery late gadolinium enhancement images with phase-sensitive reconstruction in four-chamber and two-chamber long-axis cine showing mildly heterogenous hyperenhancement. (*G* and *H*) Four-dimensional flow streamline visualization of flow acceleration through the anterior leaflet of the mitral valve in four-chamber and two-chamber long-axis cine stack of images. (*I*) Peak mitral inflow velocity trace demonstrating peak E-wave (peak mitral inflow velocity in the left ventricle in early diastole) and A-wave (peak velocity of transmitral blood flow in late diastole due to atrial contraction) velocities. (*J*) Haematoxylin and eosin stain showing classic cardiac myxoma tumoural cells with the presence of a heterogeneous matrix of tumoural cells with myxoid background forming perivascular rings, mononuclear inflammatory infiltrates surrounding small intratumoral blood vessels, and areas of thrombosis and hemosiderin-loaded macrophages. There is no cytologic atypia or mitotic activity seen.

On 4D flow two-dimensional vector visualization, during early and late filling, the blood flow accelerates through the anterior leaflet of the mitral valve. There was evidence of E/A flow reversal and absence of diastolic vortex formation (see Supplementary material online, *Video S1*). A diastolic LV vortex is formed in the low-pressure areas near the basal mitral annular area inside the LV. This doughnut-like vortex preserves the kinetic energy of blood flow during early and late filling (see Supplementary material online, *Video S2*). A lack of it would plausibly mean further loss of diastolic function and cardiac efficiency. The absence of LV diastolic vortex formation has been linked with aging and other cardiovascular diseases. The patient had successful surgical removal and confirmed histological diagnosis of myxoma (*Figure 1*).

This case highlights the importance of not only multi-parametric tissue characterization by CMR to facilitate the diagnosis of myxoma but also the use of emerging 4D flow CMR visualization and quantification tools to allow enhanced assessment of transvalvular flow and associated flow-related patterns. However, more work must be done to develop our comprehensive understanding of how vortex formation is dependent on LV geometry, pressure gradients, and mitral annulus.

## Supplementary Material

ytad298_Supplementary_Data

## Data Availability

The data underlying this article are available in the article and in its online supplementary material.

